# Prevalence and Predictors of Posting Health-Related Content Among US Facebook Users: A Cross-Sectional Survey Study

**DOI:** 10.3390/ijerph22060918

**Published:** 2025-06-10

**Authors:** Richard Bannor, Ran Xu, Jolaade Kalinowski, Tricia Leahey, Caitlin Caspi, Christie I. Idiong, Jared Goetz, Sherry Pagoto

**Affiliations:** 1 Department of Allied Health Sciences, University of Connecticut, Storrs, CT 06269-1101, USA; ran.2.xu@uconn.edu (R.X.); tricia.leahey@uconn.edu (T.L.); caitlin.caspi@uconn.edu (C.C.); christie.idiong@uconn.edu (C.I.I.); jared.goetz@uconn.edu (J.G.); sherry.pagoto@uconn.edu (S.P.); 2 Department of Human Development and Family Sciences, University of Connecticut, Storrs, CT 06269-1058, USA; jolaade.kalinowski@uconn.edu

**Keywords:** posting health-related content, Facebook and health, social media, peer-to-peer healthcare

## Abstract

Health-related content is prevalent on Facebook, but we know little about how often the typical user posts about health, the nature of such posts, or the characteristics of users who do so. We conducted a cross-sectional survey of 2508 adult US Facebook users to assess the frequency and predictors of posting about a health condition, health behavior, and health information on Facebook. The findings revealed that 68.7% of participants posted at least one type of health-related content on Facebook in the past year, and 41.6% posted all three types of health-related content. Approximately 47% posted about a health condition, 56.7% posted about health behavior, and 62.7% posted health information in the past year. Male gender, advanced education, greater Facebook engagement, having a greater number of Facebook friends, and having a chronic disease were associated with more frequent posting of all types of health-related posts (all *p*-values ≤ 0.05). Older age and longer duration on Facebook were associated with less frequent posting of all types of health-related posts (all *p*-values ≤ 0.05). Future research should explore the reasons users post health-related content on Facebook and how they evaluate the veracity of the health-related content they see and post on Facebook.

## 1. Introduction

Facebook is one of the most widely used social media platforms by US adults, with 69% of adult internet users having an account [1] and the average user spending about 31 min a day using it [2]. Health is an increasingly popular topic of discussion on Facebook. A scoping review of 53 studies examining why people talk about health on social media found that they do so to seek information about health conditions, hear about other patients’ experiences, seek support, share personal health stories, learn about treatments and side effects, and read and post reviews about doctors [3]. In 2011, the Pew Research Center was the first to report the prevalence of US adults who have gone on the internet to find and engage with others who have a similar health condition, a phenomenon they coined “peer-to-peer healthcare [4].” They found that 18% of US adults have done so, and 23% of those adults had a chronic health condition [4]. Health-related discussions on social media may be beneficial or harmful depending on the nature of those discussions [5,6]. Benefits include receipt of support and evidence-based information, which can have a positive impact on health decisions, outcomes, and quality of life, while harms include receipt of health misinformation, which could negatively impact health decisions and outcomes [6,7]. Little is known about how often the average social media user posts health-related content, the type of content they post, and the characteristics of users who do so.

Health-related posts include posts about a health behavior or a health condition, or they may involve the sharing of health information. In terms of health behavior posts, studies have examined such posts on topics including exercise [8], weight loss [9], vaccinations [10], and/or health screenings [11]. Much research on health behavior posting on social media has focused on social media platforms such as Twitter (currently X) and Instagram. A study of health-related posts on Instagram found that the most prevalent topics discussed were diet and exercise [12], which is evidence that health behavior is a common type of health post on social media. Similarly, one study analyzed over 1.5 million tweets about exercise and found that they were made primarily by weightlifters and people trying to lose weight [8]. Themes from the tweets included motivational messages and sharing or requesting information. One study surveyed 100 people who tweeted about their weight loss journey and found that they reported benefits such as social support, information exchange, and accountability [9]. Together, these studies revealed that health behavior is a common health topic of discussion on social media, and users find value in such discussions.

Research on social media posts about health conditions has largely focused on online patient communities [13,14,15,16,17,18,19] which are private online groups created for patients with a particular health condition to discuss their experiences with that condition. In 2017, a Facebook executive reported that over 70 million Facebook users participate in >6 million Facebook groups devoted to health conditions [20], numbers that surely have risen since. Studies of Facebook health groups have focused on topics such as diabetes, cancer, hypertension, mental health, genetic disorders, sexually transmitted diseases, and stroke, and findings reveal that members use these groups to share personal experiences, learn about treatments and side effects, request disease-specific guidance and feedback, and receive emotional support [21,22,23,24,25]. In 2019, in response to concerns about health information posted by users in Facebook groups being made available to advertisers, Facebook gave health groups a special designation, Health Support Group, which then came with features such as anonymous posting and a function to request that group administrators post on behalf of users [26]. Posting about one’s health condition seems to be helpful to patients on a health journey, but we do not know how prevalent this type of health posting is and how much health posting is of this type.

Finally, the third type of health post on social media is the posting of health information, which may occur in the form of links to articles about health or a post where the user states something they believe to be a fact about health. Federal health officials (e.g., Centers for Disease Control and Surgeon General) [27], health advocacy non-profit organizations [28], and healthcare professionals disseminate health information on social media, but we know little about the extent to which individual social media users share health information on social media [29]. Health information posting must be common, as several studies show that most social media users frequently see health information on social media. For example, a study of US adults during the pandemic found that 59% reported having seen information about COVID-19 on social media at least once per week, and 32% said they saw COVID-19-related content every day [30]. The same study found that 76% of participants reported that they relied on social media at least “a little” to stay informed about the pandemic, and 46% said they relied on social media “a lot” [30]. Another survey study of US adults found that participants were more likely to use social media to obtain health information for emerging infectious diseases (e.g., COVID-19) but less likely to use it to gain information about common illnesses (e.g., colds) [31]. Given the volume of health information on social media [32], how quickly health misinformation travels [33], and the negative impact of health misinformation on social media [34], research is needed to examine the prevalence of posting health information among Facebook users and the characteristics of those users.

Because people use Facebook to connect with their family, friends, and colleagues, the health content they post has the potential to influence the health-related attitudes, knowledge, and behavior of these close ties. A gap in the literature is the lack of data on the prevalence of health posts on Facebook, the type of health content being posted, and the characteristics of people who post health content. In the present study, our first aim was to determine the proportion of US adult Facebook users who have made any type of health-related post on Facebook, as well as the three types of health-related posts, including posts about health conditions, health behaviors, or health information. Our second aim was to compare the frequencies of the three types of posts. We hypothesized that Facebook users will post more about their own health, be it their health condition or health behaviors, than they post health information; this hypothesis is in line with a content analysis study of public Facebook groups on multiple sclerosis that analyzed 7029 posts that found that over 70% of posts had users talking about their experiences living with the condition [35]. Our third aim was to examine predictors of posting the three types of health-related content, including age, gender, race/ethnicity, education, Facebook engagement, number of Facebook friends, duration of Facebook use, and chronic disease status. We hypothesized that older age, female gender, higher educational level, greater Facebook engagement, greater number of Facebook friends, greater duration of Facebook usage, and having a chronic disease would be associated with posting more health-related content.

## 2. Materials and Methods

### 2.1. Study Design, Settings, and Participants

We used a cross-sectional study design and collected data from 2508 Facebook users recruited from Qualtrics survey panels. The inclusion criteria included US adults (18 to 85 years) who logged into their Facebook accounts at least one day per week. Participants responded to an anonymous survey about their Facebook use. The survey took them about 45 min to complete. We hosted the survey on Qualtrics through the University of Connecticut Office of Institutional Research site. Based on our inclusion criteria, Qualtrics identified potential participants and sent them our survey link. Participant compensation was provided through Qualtrics according to the compensation structure they have for each participant. We collected data from 11 to 19 March 2020, which was in line with when the WHO declared COVID-19 a pandemic on March 11. For dependent variables, participants were asked to reflect on their Facebook use in the past year. Informed consent was obtained from all participants. The study was conducted in accordance with the Declaration of Helsinki, and the study protocol was approved by the University of Connecticut Institutional Review Board (Project identification code: X19-055) on 12 April 2019.

### 2.2. Measures

#### 2.2.1. Independent Variables

**Demographic characteristics.** Participants were asked to indicate their age, race/ethnicity (e.g., Caucasian, Black/African American, Asian, Native Hawaiian/Other Pacific Islander, American Indian/Alaska Native, and other), gender (e.g., female, male, and non-binary/other gender), and highest education level (e.g., some high school, high school degree/GED/equivalent, trade school/specialty, some college, associate’s degree, bachelor’s degree, some graduate school, master’s degree, and doctoral degree).

**Facebook Engagement.** We asked participants how often they posted on Facebook and how often they commented on posts in the past month using a 5-point Likert scale: 0 (not at all in the past month), 1 (once in the past month), 2 (2–3 times in the past month), 3 (once a week (about 4 times in the past month)), and 4 (every day). Facebook engagement was measured using the sum of participants’ responses to posting and commenting.

**Number of Facebook friends.** We asked participants the number of Facebook friends they had.

**Duration of Facebook use.** We asked participants how long they had been on Facebook in years and months.

**Chronic disease status.** We asked participants if they had a chronic disease and, if so, to indicate which chronic disease from a list of 22 psychiatric and medical conditions, or they could enter their condition if it was not on the list.

#### 2.2.2. Dependent Variables

We measured the frequency with which participants reported posting 3 types of health-related content on Facebook in the past year: health condition, health behavior, and health information. We asked participants to indicate how often they posted about their health condition in the past year. We asked participants to indicate how often they posted about something they did for their health (for example, exercise, quit smoking, got vaccinated, got a checkup, etc.) in the past year. We also asked participants to indicate how often they posted or shared health-related information, health articles, stories, or news on Facebook in the past year. For each item, participants selected one of the responses on a 7-point Likert scale: 0 (never), 1 (once), 2 (a few times), 3 (once a month), 4 (once a week), 5 (a few times a week but not every day), and 6 (every day).

### 2.3. Data Analysis

Data analyses were conducted using Stata/SE 17.0 (StataCorp LLC, College Station, TX, USA). Descriptive statistics were used to summarize participant characteristics and the frequency of posting health-related content on Facebook. The number of Facebook friends was skewed, so we performed a log transformation [log(X + 1)]. We did not include other genders in the regression models as they formed only 0.9% (23/2508) of our sample. We compared the frequencies of posting the 3 types of health-related content on Facebook using a chi-squared test.

We conducted simple and multivariate linear regressions between the independent variables and dependent variables. We included the following independent variables: demographic characteristics (age, gender, race/ethnicity, and educational level), Facebook engagement, number of Facebook friends, duration of Facebook use, and chronic disease status as independent variables in our multivariate linear regression models. We selected these independent variables through an iterative process in line with the literature and identified significant bivariate relationships between the independent and dependent variables using the chi-squared test and correlations [36]. Regression coefficients (β) were estimated using a 95% confidence level (*p* ≤ 0.05) for all the models. We tested for multicollinearity, and all the independent variables had a variation inflationary factor of less than 2.0. Given that the dependent variables, which ranged from never (0) to every day (6), were discrete, we used Poisson (Table A1) and ordered logit (Table A2) regression models (shown in the Appendix A) to check the robustness of the results in the linear regression models.

## 3. Results

Our sample included 2508 adult Facebook users in the US. Participants were, on average, 40.9 years old (SD = 15), 74.7% were women, and 72.2% were White. About 46.7% of participants used Facebook at least once a week. Participants had a median of 290 (IQR = 101–664) Facebook friends. On average, participants had been using Facebook for 8.3 years (SD = 3.4). About one-third of participants had a chronic disease (Table 1).

A total of 47.0% reported that they posted about their health condition at least once in the past year, 56.7% posted about their health behavior, and 62.7% of participants posted health information. The majority of participants (68.7%) reported that they posted at least one of the types of health-related content in the past year, and 41.6% posted all three types of health-related content at least once in the past year (Table 2). About 38.3% reported that they posted one or more types of health-related content at least once a month. Using a chi-squared test, we found that participants posted health information more than they posted about health conditions (*p* < 0.001) and health behaviors (*p* < 0.001). However, participants posted about health behavior more than they posted about health conditions (*p* < 0.001) (Table 3).

Younger age was associated with more frequent posting about health conditions (aβ = −0.016), health behavior (aβ = −0.013), and health information (aβ = −0.011) (all *p*-values < 0.001). Men posted about health conditions (aβ = 0.479), health behavior (aβ = 0.573), and health information (aβ = 0.419) (all *p*-values < 0.001) more often than women. Race/ethnicity was not associated with any type of health-related posting. Participants with bachelor’s degrees/some graduate school education posted about health behavior (aβ = 0.200; *p* = 0.033) more often than those with a high school education. Graduate degree holders posted about health conditions (aβ = 0.587), health behavior (aβ = 0.698), and health information (aβ = 0.651) (all *p*-values < 0.001) more often than those with a high school education. Greater Facebook engagement was associated with a greater frequency of posting about health conditions (aβ = 0.274), health behaviors (aβ = 0.310), and health information (aβ = 0.306) (all *p*-values < 0.001). A greater number of Facebook friends was associated with a greater frequency of posting about health conditions (aβ = 0.072; *p* = 0.004), health behaviors (aβ = 0.121; *p* < 0.001), and health information (aβ = 0.111; *p* < 0.001). A longer duration of Facebook use was associated with less frequent posting about health conditions (aβ = −0.049), health behaviors (aβ = −0.054), and health information (aβ = −0.041) (all *p*-values < 0.001). Participants with chronic disease posted about health conditions (aβ = 0.545; *p* < 0.001), health behaviors (aβ = 0.236; *p* = 0.001), and health information (aβ = 0.357; *p* < 0.001) more frequently compared to those without a chronic disease. Of participants with chronic disease, 68.7% reported that they posted health information at least once in the past year relative to 59.8% without a chronic disease, 60.5% posted about health behavior relative to 54.8% without a chronic disease, and 58.8% posted about their health condition relative to 41.4% without a chronic disease. All the significant associations in the multivariate linear regression models were robust (i.e., aβ followed the same direction [+ or −] and *p* ≤ 0.05 in all three regression analysis methods [i.e., linear, Poisson, and ordered logit regressions]) (Table 4).

## 4. Discussion

To our knowledge, this is the first study to document the prevalence of specific types of health-related postings on Facebook (e.g., health information, health behavior, and health conditions). The results revealed that the majority (69%) of sampled Facebook users posted about health at least once in the past year, and nearly half made all three types of health-related posts. Interestingly, more than one-third posted about health regularly, i.e., at least once a month. Contrary to the hypotheses, health information was the most frequent type of health-related post, followed by health behaviors and health conditions. Men, younger adults, people with advanced degrees, people with a chronic disease, people who engage more frequently on Facebook, and people with more Facebook friends posted more frequently about health on Facebook. Taken together, these findings suggest that health appears to be a popular topic to post about on Facebook.

Findings revealed that health information was the most common type of health content posted, with 62.7% of participants saying they have posted health information in the past year. This is much higher than findings from a study using the 2007–2019 Health Information National Trends Survey (HINTS) data, in which only 17–23% of adult internet users reported having shared health information on social media sites in the past year, with little variability over that 12-year period [36]. Our study may have much higher rates because data were collected on the eve of the pandemic, when chatter about COVID-19 was beginning, and/or because our sample was composed exclusively of regular Facebook users, who might be more likely to post health information than a general sample of internet users. In the HINTS study, 14% of the sample did not use social media at all [36]. The high proportion of Facebook users posting health information raises questions about the sources of the health information users are sharing, the reasons they share health information, the health topics they post about, and the accuracy of that health information. A systematic review of the literature on health misinformation on social media found that the proportion of health-related social media posts with health misinformation ranged from 0.2% to 28.8%, depending on the health topic [37]. Posts about vaccines had the highest proportion of misinformation, whereas posts about medical treatments had the lowest [37]. In late 2020, due to the spread of health misinformation during the pandemic, Facebook moved to limit the growth of health groups by no longer showing them in recommendations [38]. Exposure to health misinformation can have detrimental impacts on health. A systematic review of 57 studies on the impact of exposure to health misinformation reported negative impacts such as negative attitudes about healthy behaviors, decreased intentions to engage in health behaviors, favorable attitudes about unhealthy behaviors, and decreased trust in health experts [34]. Research is needed to understand users’ motivations for sharing health information, the proportion of health information shared by users that is misinformation, and the impacts of sharing health information or misinformation on the user as well as on the user’s social network. Such research could benefit from theoretical models such as social media influence theory, which provides a framework for understanding how social media behavior is influenced by social network ties, how users align their social media behavior with perceived norms, and how information spreads through social networks [39].

Findings from the current study revealed that over half of the sampled Facebook users (56.7%) posted about their health behavior. Although no studies have examined the prevalence of health behavior posting on social media, previous research has documented specific types of health behavior posted about on social media, with findings showing that diet and physical activity are popular topics [12]. Social media users who post about their health behaviors cite social support, accountability, and positive reinforcement as reasons they do so [40]. The term or hashtag, “fitspiration”, has been coined to refer to posting about having just worked out [41]. Studies are mixed as to whether such posts have a positive or negative impact on the users’ followers, and it may very much depend on the content of the post, including the images [42,43,44]. Some studies have found that exposure to fitspiration posts may reduce body satisfaction and increase negative mood among women when images in the posts reinforce unrealistic beauty ideals [42,43]. On the other hand, a study of 485 social media users found that exposure to fitspiration content from close ties on social media was related to greater positive attitudes about exercise enjoyment and higher descriptive social norms [44]. Possible negative impacts may occur when the health behavior being posted about sounds healthy but is not actually healthy. A systematic literature review showed that social influence has a strong impact on the adoption of fad diets and unhealthy weight control behaviors [45]. Social comparison theory suggests that people assess their own worth based on comparisons they make with others [46]. Social media has the potential to amplify social comparison by exposing users to a large social network and repeated messaging about health and fitness. Future research is needed to understand the characteristics of health behavior posts that social media users find motivating versus demoralizing, as well as those that may encourage the adoption of unhealthy versus healthy behaviors.

The present study revealed that nearly half of the sampled users (47%) have posted about their health conditions, and not surprisingly, those with chronic conditions were more likely to post about their health conditions (58.8% vs. 41.4%). This provides an update to the 2011 Pew Research Center data that showed that 18% of US adult internet users and 23% of those with a chronic disease have gone online to find others with their health condition [47]. Although posting about one’s health on Facebook is becoming increasingly common, patients’ desires to exchange their health stories are not a new phenomenon. Patient narratives have been found to provide information for patients about their disease journey [48] to improve coping [49], to identify questions to ask healthcare providers [50], to increase healthcare participation while decreasing unnecessary healthcare utilization, and to inform healthcare decision-making [50]. According to transportation theory, narratives can have a persuasive impact, thereby influencing the behavior of those who identify with those narratives [51]. Social media platforms, patient blogs, and other patient forums may increase the impact of patient narratives given their reach, and they are being increasingly used by patients to learn not only about their health condition but to hear directly from patients living with the condition [48]. The finding that a shorter duration of Facebook use was associated with more health posting might be a sign that health conversations and communities may be drawing new users to Facebook. Given that so many patients seek to share and/or consume patient narratives as they embark on a healthcare journey, research is needed to explore ways this can be integrated into the healthcare experience. Further research is also needed to understand what Facebook users share about their health conditions on Facebook, the extent to which such postings are to their friend network or in private health groups, and how this behavior impacts their health status.

Contrary to our hypothesis, we found that men reported posting more health-related content compared to women. This finding is inconsistent with the findings of the study that used the 2019 HINTS data, which found that women were twice as likely to post health content on social media compared to men [36]. Our study recruited only Facebook users, but the HINTS sample included internet users who may or may not use social media [36], which may account for the differences observed. Our findings are also inconsistent with a study that found that women made over half (56%) of the posts in private Facebook groups for Arabic-speaking people with diabetes. Women may feel more comfortable discussing health in private online spaces. Two other studies examined health content on Facebook but did not report the gender of the users who made health-related posts [35,52]. More research is needed to understand gender differences in posting health content on Facebook, including whether men and women post different types of health content and where each is most likely to have health discussions (private groups vs. public spaces vs. personal profile).

### Limitations

This study has some limitations that should be considered. One limitation is that our sample focused on US adult Facebook users who log into Facebook one or more days a week. As such, these findings may not be generalizable to all Facebook users in the US or users in other countries. Women were overrepresented in our sample (approximately 75%), whereas 54% of Facebook users in the US are women [53]. Another limitation is that the number of users who posted health-related content and the frequency of health-related posts that were made may have been heightened by social media discussions about COVID-19 because we collected data in March 2020 and shortly after the WHO declaration of the COVID-19 pandemic [54,55]. Participants were asked to reflect on their health posting in the past year; however, health-related posting likely increased starting in the weeks before the pandemic, which may have created a recency effect that inflated estimates of health posting in the past year. Health-related posts likely increased even further in 2020 through 2022 as the pandemic continued to evolve. Longitudinal studies should examine the trajectory of health posting over longer periods of time to examine the influence of current events on health-related discussions.

## 5. Conclusions

Our study extends the Pew Research Center’s 2011 report, showing that 18% of internet users have used social media to discuss health [4]. Our findings revealed that by 2020, nearly 69% of Facebook users had posted health-related content, and one-third reported doing so on a fairly regular basis. This suggests that health is a popular topic on Facebook, which has implications for public health organizations and health communication researchers. Public health organizations that disseminate health messaging may find an audience for that content on Facebook; however, research is needed to determine the nature of public health content that Facebook users are willing to disseminate, as well as their willingness to share content from various sources (e.g., non-profit organization vs. peer vs. commercial vs. government organization). Further research is also needed to understand where users obtain the health information they share (e.g., government, healthcare professionals, peers, media, or disinformants), whether people put their privacy at risk when sharing certain types of personal health information (e.g., lab reports), the impact of the health information they share on their social media followers, the reasons they post health-related content, the veracity of the health content they post, the presence of health misinformation in their posts, and the type of posts most likely to contain health misinformation. Theoretical models, including social media influence theory [39], transportation theory [51], and social comparison theory [46], can be used to guide such research. Future research should also more regularly examine the prevalence and type of health posting among users of all social media platforms, as this likely changes over time, particularly during major public health crises. Healthcare providers, public health organizations, and governmental agencies globally should use social media platforms to disseminate evidence-based health information and correct and counter health misinformation [56,57].

## Figures and Tables

**Table 1:** Demographic characteristics of adult Facebook users (N = 2508).

	N (%) or M (±SD)
Age in years	40.9 (±15.0)
Gender	
Women	1874 (74.7)
Men	611 (24.4)
Other	23 (0.9)
Race/ethnicity	
Non-Hispanic White	1810 (72.2)
Black/African American	266 (10.6)
Hispanic	208 (8.3)
Other	224 (8.9)
Highest educational level	
Less than high school/high school degree/GED/equivalent	661 (26.4)
Trade/technical/some college/associate’s degree	1005 (40.1)
Bachelor’s degree/some graduate school	526 (21.0)
Master’s degree/doctoral degree	316 (12.6)
Facebook engagement	
Not at all in the past month	158 (6.3)
Once in the past month	509 (20.3)
Less than once a week (2–3 times)	670 (26.7)
Once a week (about 4 times)	573 (22.9)
Every day	598 (23.8)
Number of Facebook friends (median; IQR)	290 (101–664)
Number of Facebook friends ^a^	5.5 (±1.5)
Duration of Facebook use (years)	8.3 (±3.4)
Chronic disease status	
No	1691 (67.4)
Yes	817 (32.6)

Number of Facebook friends ^a^ = log-transformed [log(X + 1)].

**Table 2:** Proportion of participants posting health-related content on Facebook (N = 2508).

Posting Health-Related Content on Facebook	n (%)
Never posted any type of health-related content	784 (31.3)
Posted at least one type of health-related content	1724 (68.7)
Posted only one type of health-related content	318 (12.7)
Posted two types of health-related content	363 (14.5)
Posted all three types of health-related content	1043 (41.6)

**Table 3:** Distribution of posting health-related content on Facebook among adult Facebook users (N = 2508).

	Posting Health Condition N (%)	Posting Health Behavior N (%)	Posting Health Information N (%)	*p*-Value
				all *p*-Values < 0.001
Never	1328 (52.9)	1087 (43.3)	936 (37.3)	
Once	196 (7.8)	225 (9.0)	237 (9.5)	
A few times	412 (16.4)	507 (20.2)	551 (22.0)	
Once a month	174 (6.9)	233 (9.3)	243 (9.7)	
Once a week	152 (6.1)	182 (7.3)	234 (9.3)	
A few times a week	157 (6.3)	165 (6.6)	196 (7.8)	
Every day	89 (3.6)	109 (4.3)	111 (4.4)	

**Table 4:** Linear regression of predictors of posting health-related content on Facebook among adult Facebook users.

	Crude						Multivariate					
	Posting Health Condition		Posting Health Behavior		Posting Health Information		Posting Health Condition		Posting Health Behavior		Posting Health Information	
	β	*p*-Value	β	*p*-Value			aβ	*p*-Value	aβ	*p*-Value	aβ	*p*-Value
Age in years	−0.017 ^R^	<0.001	−0.018 ^R^	<0.001	−0.015 ^R^	<0.001	−0.016 ^R^	<0.001	−0.013 ^R^	<0.001	−0.011 ^R^	<0.001
Gender												
Women	0	Ref.	0	Ref.	0	Ref.	0	Ref.	0	Ref.	0	Ref.
Men	0.711 ^R^	<0.001	0.872 ^R^	<0.001	0.697 ^R^	<0.001	0.479 ^R^	<0.001	0.573 ^R^	<0.001	0.419 ^R^	<0.001
Race/ethnicity												
Non-Hispanic White	0	Ref.	0	Ref.	0	Ref.	0	Ref.	0	Ref.	0	Ref.
Black/African American	0.344 ^R^	0.004	0.358 ^R^	0.003	0.295 ^R^	0.016	0.146	0.169	0.095	0.364	0.087	0.419
Hispanic	0.298	0.024	0.427 ^R^	0.001	0.274 ^R^	0.044	0.077	0.512	0.155	0.184	0.057	0.634
Other	0.215	0.092	0.178	0.171	0.180	0.170	0.151	0.190	0.097	0.393	0.132	0.259
Highest educational level												
Less than high school/high school	0	Ref.	0	Ref.	0	Ref.	0	Ref.	0	Ref.	0	Ref.
Trade/technical/some college/associate’s degree	−0.135	0.130	−0.116	0.199	−0.119	0.193	0.014	0.861	0.051	0.516	0.020	0.810
Bachelor’s degree/some graduate school	−0.223 ^R^	0.032	0.032	0.762	−0.070	0.513	−0.026	0.782	0.200 ^R^	0.033	0.097	0.315
Master’s degree/doctoral degree	0.757 ^R^	<0.001	0.982 ^R^	<0.001	0.910 ^R^	<0.001	0.587 ^R^	<0.001	0.698 ^R^	<0.001	0.651 ^R^	<0.001
Facebook engagement	0.303 ^R^	<0.001	0.342 ^R^	<0.001	0.338 ^R^	<0.001	0.274 ^R^	<0.001	0.310 ^R^	<0.001	0.306 ^R^	<0.001
Number of Facebook friends ^a^	0.216 ^R^	<0.001	0.286 ^R^	<0.001	0.267 ^R^	<0.001	0.072 ^R^	0.004	0.121 ^R^	<0.001	0.111 ^R^	<0.001
Duration of Facebook use (years)	−0.023	0.031	−0.019	0.075	−0.006	0.559	−0.049 ^R^	<0.001	−0.054 ^R^	<0.001	−0.041 ^R^	<0.001
Chronic disease status												
No	0	Ref.	0	Ref.	0	Ref.	0	Ref.	0	Ref.	0	Ref.
Yes	0.392 ^R^	<0.001	0.079	0.311	0.222 ^R^	0.005	0.545 ^R^	<0.001	0.236 ^R^	0.001	0.357 ^R^	<0.001

Number of Facebook friends ^a^ = log-transformed [log(X + 1)]. β = crude regression coefficient; aβ = multivariate regression coefficient; ^R^ = robust association (i.e., β or aβ follows the same direction [+ or −] and *p* ≤ 0.05 in all three regression analysis methods).

## Data Availability

The data presented in this study are available on request from the corresponding author. (The data are not publicly available due to privacy or ethical restrictions.).

## Appendix A

In these Appendices, we included the Poisson and Ordered logit regression models (both crude and multivariate) of predictors of posting health-related content on Facebook among adult Facebook users. We included these regression models to check the robustness of the results in the linear regression models in the main text.

**Table A1:** Poisson regression of predictors of posting health-related content on Facebook among adult Facebook users.

	Crude						Multivariate					
	Posting Health Condition		Posting Health Behavior		Posting Health Information		Posting Health Condition		Posting Health Behavior		Posting Health Information	
	β	*p*-Value	β	*p*-Value	β	*p*-Value	aβ	*p*-Value	aβ	*p*-Value	aβ	*p*-Value
Age in years	−0.013	<0.001	−0.011	<0.001	−0.008	<0.001	−0.014	<0.001	−0.010	<0.001	−0.007	<0.001
Gender												
Women	0	Ref.	0	Ref.	0	Ref.	0	Ref.	0	Ref.	0	Ref.
Men	0.463	<0.001	0.474	<0.001	0.346	<0.001	0.283	<0.001	0.273	<0.001	0.181	<0.001
Race/ethnicity												
Non-Hispanic White	0	Ref.	0	Ref.	0	Ref.	0	Ref.	0	Ref.	0	Ref.
Black/African American	0.234	<0.001	0.206	<0.001	0.153	0.001	0.095	0.078	0.050	0.319	0.039	0.413
Hispanic	0.206	<0.001	0.241	<0.001	0.143	0.006	0.044	0.466	0.075	0.163	0.024	0.653
Other	0.153	0.008	0.108	0.046	0.096	0.058	0.108	0.072	0.061	0.272	0.070	0.179
Highest educational level												
Less than high school/high school	0	Ref.	0	Ref.	0	Ref.	0	Ref.	0	Ref.	0	Ref.
Trade/technical/some college/associate’s degree	−0.102	0.018	−0.077	0.058	−0.068	0.070	0.012	0.793	0.026	0.528	0.008	0.819
Bachelor’s degree/some graduate school	−0.175	0.001	0.020	0.664	−0.040	0.369	−0.027	0.620	0.120	0.012	0.053	0.242
Master’s degree/doctoral degree	0.435	<0.001	0.486	<0.001	0.409	<0.001	0.321	<0.001	0.321	<0.001	0.275	<0.001
Facebook engagement	0.234	<0.001	0.220	<0.001	0.191	<0.001	0.212	<0.001	0.199	<0.001	0.173	<0.001
Number of Facebook friends ^a^	0.164	<0.001	0.182	<0.001	0.150	<0.001	0.047	<0.001	0.066	<0.001	0.056	<0.001
Duration of Facebook use (years)	−0.016	0.001	−0.012	0.011	−0.003	0.425	−0.029	<0.001	−0.026	<0.001	−0.018	<0.001
Chronic disease status												
No	0	Ref.	0	Ref.	0	Ref.	0	Ref.	0	Ref.	0	Ref.
Yes	0.272	<0.001	0.048	0.147	0.117	<0.001	0.383	<0.001	0.147	<0.001	0.180	0.002

Number of Facebook friends ^a^ = log-transformed [log(X + 1)]. aβ = multivariate regression coefficient.

**Table A2:** Ordered logit regression of predictors of posting health-related content on Facebook among adult Facebook users.

	Crude						Multivariate					
	Posting Health Condition		Posting Health Behavior		Posting Health Information		Posting Health Condition		Posting Health Behavior		Posting Health Information	
	β	*p*-Value	β	*p*-Value	β	*p*-Value	aβ	*p*-Value	aβ	*p*-Value	aβ	*p*-Value
Age in years	−0.017	<0.001	−0.019	<0.001	−0.016	<0.001	−0.022	<0.001	−0.020	<0.001	−0.015	<0.001
Gender												
Female	0	Ref.	0	Ref.	0	Ref.	0	Ref.	0	Ref.	0	Ref.
Male	0.656	<0.001	0.815	<0.001	0.640	<0.001	0.558	<0.001	0.654	<0.001	0.456	<0.001
Race/ethnicity												
Non-Hispanic White	0	Ref.	0	Ref.	0	Ref.	0	Ref.	0	Ref.	0	Ref.
Black/African American	0.333	0.006	0.378	0.001	0.300	0.010	0.115	0.385	0.061	0.621	0.035	0.778
Hispanic	0.221	0.113	0.371	0.006	0.246	0.063	0.005	0.974	0.115	0.419	0.068	0.622
Other	0.219	0.097	0.179	0.165	0.186	0.140	0.166	0.248	0.084	0.548	0.149	0.269
Highest educational level												
Less than high school/high school	0	Ref.	0	Ref.	0	Ref.	0	Ref.	0	Ref.	0	Ref.
Trade/technical/some college/associate’s degree	−0.114	0.221	−0.092	0.314	−0.122	0.173	0.024	0.810	0.090	0.363	0.011	0.907
Bachelor’s degree/some graduate school	−0.305	0.007	0.041	0.699	−0.072	0.494	−0.078	0.525	0.317	0.006	0.123	0.270
Master’s degree/doctoral degree	0.644	<0.001	0.917	<0.001	0.844	<0.001	0.368	<0.001	0.792	<0.001	0.663	<0.001
Facebook engagement	0.364	<0.001	0.395	<0.001	0.376	<0.001	0.353	<0.001	0.384	<0.001	0.353	<0.001
Number of Facebook friends ^a^	0.242	<0.001	0.304	<0.001	0.284	<0.001	0.101	0.001	0.152	<0.001	0.138	<0.001
Duration of Facebook use (years)	−0.013	0.258	−0.013	0.206	−0.001	0.921	−0.055	<0.001	−0.066	<0.001	−0.046	<0.001
Chronic disease status												
No	0	Ref.	0	Ref.	0	Ref.	0	Ref.	0	Ref.	0	Ref.
Yes	0.525	<0.001	0.122	0.113	0.255	0.001	0.816	<0.001	0.369	<0.001	0.434	<0.001

Number of Facebook friends ^a^ = log-transformed [log(X + 1)]. aβ = multivariate regression coefficient.
